# Unlocking participation: factors associated with attendance on a voluntary domestic abuse perpetrator programme

**DOI:** 10.3389/fsoc.2026.1787740

**Published:** 2026-06-19

**Authors:** Nathan Eisenstadt, Daisy Gaunt, Rwth Leach, Karen Morgan, Gene Feder, Helen Cramer

**Affiliations:** Bristol Medical School, Centre for Academic Primary Care, University of Bristol, Bristol, United Kingdom

**Keywords:** attendance, attrition, domestic abuse, drop out, perpetrator programme, behavior change

## Abstract

**Introduction:**

Domestic abuse perpetrator programmes (DAPPs) show evidence of reducing recidivism, but study size, quality, varying participant risk levels, and intervention length have all contributed to limited effect sizes. A significant challenge for both the effectiveness of DAPPs and the measurement of that effectiveness is high treatment attrition. Despite a growing body of research, there remains uncertainty about which participant characteristics predict drop-out versus completion, and most existing evidence focuses on court-mandated rather than voluntary programmes.

**Methods:**

This paper analyses factors associated with attendance on REPROVIDE, a voluntary 23-week DAPP for men in relationships with women. Referral routes, perpetrator demographics, intrapersonal characteristics, and violence-related variables were examined using number of sessions attended as a continuous dependent variable. Univariate linear regression analyses were conducted first, followed by multivariable linear regression incorporating age, income, PHQ-9 score, income reporting status, and number of pre-group assessment sessions attended. All predictors were measured at baseline.

**Results:**

Univariate analyses identified several factors associated with higher session attendance: number of pre-group assessment sessions attended, older age, greater depression severity (higher PHQ-9), higher reflective functioning (higher RFQ-6), self-referral, employment, and higher income. Lower attendance was associated with refusal to disclose income, involvement of Child and Family Court or Children's Social Care, and referral by health, criminal justice, or social services. The multivariable model showed evidence of an association with number of group sessions attended, with an adjusted *R*^2^ of 0.1696.

**Discussion:**

Greater clarity on the factors associated with men's voluntary DAPP attendance has practical implications for both researchers and practitioners. For researchers, these findings can inform the design and testing of future interventions. For practitioners, they can guide resource allocation and process design aimed at maximising treatment adherence.

## Introduction

Domestic abuse (DA) is a violation of human rights that damages individual health and wellbeing, with a heavy socio-economic impact. Domestic abuse perpetrator programmes (DAPPs) are a critical component in the prevention of domestic abuse. With a core focus on victim/survivor safety, DAPPs seek to enable those who perpetrate abusive behaviors to stop. Recent meta-analyses of the effectiveness of DAPPs at reducing DA recidivism show mixed results, with study size, quality, and differing within-study participant risk levels contributing to limited effect sizes (Arce et al., 2020; Nesset et al., 2019; Travers et al., 2021; Babcock et al., 2024; Vall et al., 2021). A key challenge for the measurement of the effectiveness of DAPPs is treatment dropout which is consistently high, ranging from 15 to 58% across multiple studies (Santirso et al., 2020a, p. 176). If those who do not benefit or are “harder to treat” drop out of the intervention (and, crucially, the study), effect sizes may be overestimated while if those who improve quickly drop out, effect sizes may be underestimated. Intention to treat protocols (ITT) which include all participants assigned to the treatment group address this to some degree but tend to show more conservative effect sizes, in part due to attrition. Under ITT, if those who drop out of the intervention remain in the study (completing questionnaires on their behavior) the effectiveness of the intervention is measured in part using data from those who did not complete the intervention—and may never have started it. Conversely, if those who drop out of the intervention also drop out of the study, sample size is reduced, limiting the ability of researchers to detect an effect and/or conduct sub-group analyses.

Dropout also presents challenges for DAPP delivery/practice (i.e., outside of research studies). Dropout has been shown to predict domestic abuse reoccurrence (Lila et al., 2019) and many of the factors associated with domestic abuse reoccurrence are also associated with dropout (Jewell and Wormith, 2010). Meanwhile, short interventions have failed to reduce recidivism and may have negative effects (Arce et al., 2020) suggesting that participant retention on longer interventions may be beneficial. More practically, participant retention is extremely time-consuming for practitioners diverting resources away from other avenues of potential harm reduction and may indicate that the intervention is not suited/acceptable to a particular cohort. Dropout may also limit the potential positive impact of interventions which have already demonstrated effectiveness since fewer people are attending sufficient sessions needed to change their behavior. This last claim only holds, of course, where it is known that those who drop out could have benefited—underlining the need to know more about who drops out and why. Understanding the factors that predict treatment attrition is thus of critical importance to DAPP researchers and practitioners alike.

Despite the importance of understanding treatment attrition, it is often not measured or is measured inconsistently in evaluations and trials of DAPPs (Johnston et al., 2026; Lilley-Walker et al., 2018) and where attrition is reported, and among studies analysing treatment attrition specifically, the evidence as to which factors predict dropout or completion is mixed and, in places, contradictory.

Factors associated with programme (treatment) completion have included being older (Askeland and Heir, 2013; Cunha et al., 2023; Lauch et al., 2017; Priester et al., 2019; Scott et al., 2013), more educated (Priester et al., 2019; Trebow et al., 2015), being white (in majority-white societies) (Chang and Saunders, 2002; Eckhardt C. et al., 2008; Lauch et al., 2017; Priester et al., 2019; Taft et al., 2001a), being married or having a long term relationship (Bowen and Gilchrist, 2006; Buttell and Carney, 2008; Carney et al., 2006), having children (Rondeau et al., 2001), being court-mandated to attend (Buttell and Carney, 2008; Buttell and Pike, 2002; Lauch et al., 2017; Scott, 2004; Taft et al., 2001b).

Other studies have found no significant effect on programme completion with age (Buttell and Pike, 2002), education level (Buttell and Carney, 2008; Buttell and Pike, 2002; Cunha et al., 2023) ethnicity (Bennett et al., 2010; Cunha et al., 2023; Stoops et al., 2010) relationship status (Buttell and Carney, 2008; Chang and Saunders, 2002; Cunha et al., 2023) and being court-mandated (Cunha et al., 2023; Duplantis et al., 2006). Against the prevailing trend in the literature and albeit in older studies, (Daly et al., 2001) found that completers had more prior arrests or convictions while (Gerlock, 2001) found completers to be younger.

Associations with dropout have included having a criminal history (Bennett et al., 2010; Bowen and Gilchrist, 2006; Cantos et al., 2015; Richards et al., 2021), having substance use problems (Buttell and Carney, 2008; Lila et al., 2020; Rondeau et al., 2001), having alcohol and/or substance use problems and trauma (Expósito-Álvarez et al., 2025), having antisocial traits (Chang and Saunders, 2002; Rock et al., 2013; Romero-Martínez et al., 2021), borderline personality disorder (Munro and Sellbom, 2022), and scoring high on the MMPI-2-RF (measuring personality disorders and psychopathology) (Whitman et al., 2020) or State-Trait Anger Expression Inventory (Eckhardt C. I. et al., 2008); having attention deficit hyperactivity disorder (ADHD; Romero-Martínez et al., 2023b) and having experienced abuse as a child (Chang and Saunders, 2002; Lauch et al., 2017; Priester et al., 2019).

Others have found no significant effect on completion with substance misuse histories (Buttell and Carney, 2002, 2008; Buttell and Pike, 2002; Chang and Saunders, 2002; Stoops et al., 2010) or having experienced abuse as children (Cuevas and Bui, 2016; Rondeau et al., 2001). No effect, of course, simply means “no evidence of an association” which can be challenging to detect for the same reasons that render effectiveness of DAPPs challenging to demonstrate. Lack of agreement, however, may have more to do with differences in measurement and programme types including content/approach, cohorts, duration, voluntary vs. compulsory referral routes and/or local/country contexts (Jewell and Wormith, 2010). *Dropout* and *completion* are themselves contested concepts with different studies defining them more or less “strictly”. As (Jewell and Wormith 2010) note, (Beldin 2008) categorize “dropouts” as those who miss one or sessions whereas (DeHart et al. 1999) place the threshold at 67% attendance. More recently, (Cunha et al. 2023) define “dropouts” as those who attend an initial assessment but complete less than 75% of sessions and “completers” as those who attend at least 75%. Cunha et al. thus include what some refer to as “no shows” in their definition of dropouts—those participants who attend an initial assessment but do not attend a single treatment session, whereas others exclude these participants. This can make it difficult to tell whether the factors that predict initial acceptance are different from those which predict longer-term engagement (Richards et al., 2021). Comparing “no shows”, with those who dropped out in-treatment and those who completed, (Richards et al. 2021) found “no shows” to have the highest levels of risk factors for DA perpetration and lowest levels of protective factors followed by drop-outs then completers.

The literature is mixed in terms of whether researchers use binary, categorical or continuous variables to measure correlates of attendance. Using binary variables entails setting a number of sessions which count as “complete” and a (lower) number of sessions (or zero sessions) which counts as “incomplete” or “dropped out”. This enables clear claims about “dropouts” vs. “completers” but limits the degree to which comparisons can be made across studies due to difference in how strictly dropout/completion is defined, example, what percentage of sessions missed count a participant as a “dropout” (Jewell and Wormith, 2010). Interventions also vary in length with longer interventions offering more opportunities to drop out (Bell and Coates, 2022; Daly and Pelowski, 2000) and thus shorter interventions appearing “better” at limiting attrition (Cissner and Puffett, 2006). Measuring attrition using a continuous variable (number of sessions attended rather than categories of attendance; e.g., Rooney and Hanson, 2001) avoids the imposition of arbitrary categories but is not amenable to succinct claims about “dropouts” and “completers” as distinct groups.

It is also the case that existing studies of attrition, tend to look either at court-mandated programmes or programmes which accept both court-mandated and voluntary participants. In Cunha et al.'s (2024) review, only three programmes are voluntary and of those only one uses a group format alongside individual treatment (Askeland and Heir, 2013). Given the different risk profiles, levels of offending and motivations/readiness to change of perpetrators on voluntary, vs. court-mandated DAPPs (Bowen and Gilchrist, 2004), it is likely that the factors which predict attendance may be quite different. Understanding the factors associated with treatment attrition on voluntary DAPPs is especially important, precisely because of their voluntary nature—there are fewer and less powerful avenues for compelling engagement/sanctioning disengagement. Voluntary DAPPs may play a critical role in prevention helping men to change *before* their behavior escalates causing victims and survivors additional harms and generating significant preventable costs to health, social care and criminal justice and systems.

Fitz-Gibbon et al.'s (2024) review of factors associated with dropout and completion note that completion is higher on targeted programmes tailored to specific populations and highlights the importance of programme readiness and individualized support. In order to understand whether and/or how voluntary DAPPs in particular, may be targeted to specific populations, and how those who struggle to attend may be better supported, more research is needed on the factors which facilitate or inhibit attendance on this type of group programme. To this end, in this study we aimed to see if there any patterns in the attendance data that show a correlation between participant type (demographics, referral route, relationship status, and/or other factors) and the likelihood that participants will complete a greater or lower number of group sessions on a voluntary DAPP.

## Intervention description

The data used here are from the REPROVIDE Trial—a randomized controlled trial of a voluntary, group-based domestic abuse perpetrator programme (DAPP). Eligible participants were men over 21 years of age who admitted to some abusive behavior toward a female partner or ex-partner. Men who were court-mandated to attend a DAPP and/or had ongoing criminal justice and/or ongoing or recently closed (within the last 12 months) Child and Family Court proceedings were ineligible. Given inconsistent evidence on who might benefit most and/or be most likely to attend such a group intervention, other eligibility criteria were broad. Alcohol/substance use and/or mental ill-health severity were not exclusion criteria unless it was deemed by assessors at recruitment that a participant would be unable to attend and meaningfully participate in the group. Given the exclusion of court-mandated perpetrators, the intended cohort were broadly low-medium risk. Risk level was assessed by expert practitioner judgement prior to randomisation and higher-risk men were excluded case by case. Participant intake was on a rolling basis with monthly intakes.^1^

Programme content was designed by Chris Newman and Kate Iwi for and in partnership with Respect—an umbrella body and accrediting organization for domestic abuse perpetrator work in the UK. The programme was a manualised, “hybrid” DAPP, combining trauma-informed and gendered-psychoeducational (or “Duluth”) approaches with elements of cognitive behavioral therapy (CBT) and motivational interviewing (MI) techniques. Facilitators were trained in the programme, but formal qualifications (e.g., clinical or therapeutic) were not required—this is in line with prevailing practice in the UK. Following Respect practice guidelines, support for intervention men's female ex-/partners was integrated with the DAPP provision. Due to the present study being on DAPP intervention attrition, only data from intervention men is presented in here.

The intervention consisted of 23 manualised group sessions with an allowance for two additional non-manualised sessions to be added in preparation for and to debrief after the winter holidays should the intervention run over this period. This is due to the winter holidays being recognized as a higher risk time-period for domestic abuse in the UK. Some delivery sites added up to two sessions in addition to these pre- and post-holiday sessions based on practitioner assessment of need. In addition to the group sessions, and as part of the intervention design, men were able to attend pre-group one-to-one sessions with a practitioner. The aim of these was to prepare a man for the DAPP group and to provide practitioners with additional information for risk management. In practice, the number of pre-group one to one sessions provided ranged from 0 to 6 due to a combination of practitioner judgement of men's need, and men's willingness to attend these sessions.

On REPROVIDE, 52% of the intervention group completed fewer than 23 group sessions. However, even with this level of attrition, and as reported in the main trial paper (Cramer et al. forthcoming), the intervention was effective in reducing men's self-reported ABI scores in comparison to the control group (actual point estimates and 95% CI). Crucially for the present study, sensitivity analyses revealed that REPROVIDE was *more* effective for participants who attended 12–27 group sessions (actual point estimates and 95% CI). Given that we know (a) the intervention is effective and (b) that it is more effective for men who attended above a certain threshold of group sessions, it is critical to know more about those who dropped out and why, and conversely, about which factors predict or are correlated with attendence at greater or fewer group sessions.

## Methods

Using baseline pre-randomisation data from 161 participants (of 206 randomized minus 45 where data was missing) involved in the active treatment group of the REPROVIDE trial (Morgan et al., 2023), we tested the association between the continuous variable of number of treatment sessions attended and the following participant characteristics using linear regression models: age, ethnicity, highest qualifications, income, employment status, relationship status, self-reported children's social care (CSC) or child and family court (CAFCASS) involvement, self-reported criminal justice involvement, experienced/witnessed domestic abuse or sexual violence as a child, alcohol misuse (AUDIT 3) illicit substance use, depression severity (PhQ-9 score), abusive behavior reported (revised ABI-29), autism (AQ-10) and reflective functioning (RFQ-6). Further detail on these measures is available in the protocol paper (Morgan et al., 2023). In addition to this, number of post-randomisation, pre-group one-to-one sessions were included in the analysis since it was theorized that additional practitioner contact prior to the first session attended may increase treatment adherence.

Initially baseline data were explored in univariate analyses. For those that showed evidence of association with number of treatment sessions, we used a multivariable linear regression model to further investigate the potential associations. This was to explore the combination of factors that were most strongly associated with attendance, and reduce confounding or correlation between factors. This included the variables of PHQ-9, age, income reporting (yes/no), income and number of pre-assessment sessions attended. Exploratory univariate analyses were also conducted to investigate potential associations between income report (binary) and RFQ-6 score, and criminal justice involvement (binary), using logistic regression models. Other univariate analyses logistic regression models were conducted to explore the potential associations between CSC/CAFCASS involvement (binary) and: PHQ-9 score, RFQ-6 score, ABI score, AUDIT score, PTSD (binary).

Due to variance in definitions of dropout and completion at the intervention sites and, as discussed above, within the literature, session attendance as a continuous variable was used in the analysis.

## Results

**Figure 1:**
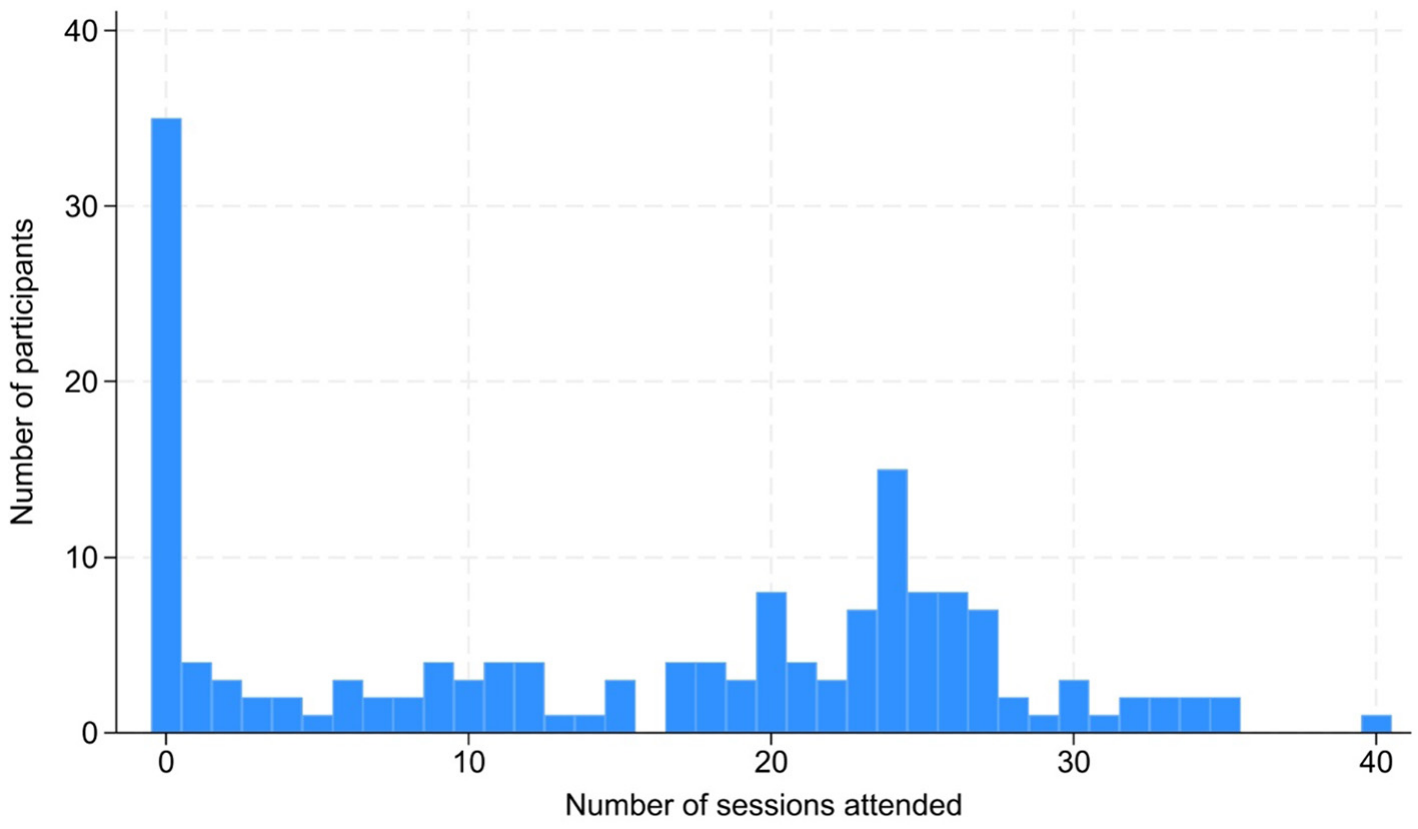
Number of sessions attended.

One hundred and twenty-six men attended at least one intervention session, with the median number of sessions attended being 22 (IQR 1, 34). Thirty-five men (22% of 161) attended 0 sessions. 61 men (48%) attended 23 or more sessions so can be understood as having ‘completed' the intervention by this metric. Eighty-three men (66%) attended at least 18 sessions (80% of the intended minimum number of sessions in the full intervention). The distribution of sessions attended is shown in Figure 1.

### Socio demographic data

Table 1 shows that the average age of men was 38 years, with the majority identifying as White. Education-levels were lower than National averages with 14% reporting Level 4 or above [compared to 33.8% in England and Wales in 2021 (Office for National Statistics, 2021)]. 55% were employed [compared to 74.5% the UK (Office for National Statistics, 2024)], with 17% declaring earnings under £12,000.^2^

**Table 1:** Baseline demographics, mental health, alcohol, substance use and violence and motivation related variables.

Variable	n/N (%) or mean (standard deviation, *n*)
Age	38.47 (10.98), *n* = 161
Ethnicity	
White	139/161 (86%)
Asian or Asian British	3/161 (2%)
Black/African/Caribbean/Black British	3/161(2%)
Mixed	9/161 (6%)
Other ethnic group	4/161 (2%)
Unknown	3/161 (2%)
Highest education qualification achieved	
No qualifications	35/158 (22%)
O-levels/GCSEs	25/158 (16%)
NVQ levels 1–3/GNVQ	22/158 (14%)
A-levels	8/158 (5%)
NVQ levels 4–5/HNC HND	8/158 (5%)
Degree/higher degree	14/158 (9%)
Other	46/158 (29%)
Employment	
Employed	88/159 (55%)
Looking after home/family	9/159 (6%)
Unemployed and looking for work	20/159 (13%)
Unable to work due to long term sickness	21/159 (13%)
Retired from paid work	5/159 (3%)
In full time education	1/159 (1%)
Other	15/159 (9%)
Income	
Up to £5,000	13/158 (8%)
£5,000–£11,999	14/158 (9%)
£12,000–£21,999	32/158 (20%)
£22,000–£37,999	42/158 (27%)
£38,000 to £72,000, and above	30/158 (19%)
Prefer not to say/don't know	27/158 (17%)
Has children	132/156 (84%)
Self-reported criminal justice involvement in last 12 months	78/157 (50%)
Self-reported CSC/CAFCASS involvement if children under 18	76/132 (58%)
Relationship status	
Together and living together	49/159 (31%)
Together but living apart	28/159 (18%)
In the process of splitting up	1/159 (1%)
The relationship has ended and we are living apart with no contact	28/159 (18%)
The relationship has ended and we are living apart and still have contact	29/159 (18%)
I am not sure	17/159 (11%)
Something else	7/159 (4%)
Relationship hopes	
That we will be together and living together	100/159 (63%)
That this relationship will end	4/159 (3%)
I am not sure	21/159 (13%)
I am in another relationship already	6/159 (4%)
Something else	28/159 (18%)
ABI-29 score	43.61 (12.03), *n* = 159
Experienced or witnessed abuse as a child	107/158 (68%)
PTSD: yes	82/159 (52%)
AUDIT score	3.99 (3.37), *n* = 154
Illicit drug use: yes	38/159 (24%)
PHQ-9 score	10.88 (6.20), *n* = 159
AQ-10 autism score	4.31 (2.07), *n* = 156
AQ-10 clinical cut-off (6+)	42/156 (27%)
RFQ-6	4.62 (1.39), *n* = 156
Referral source	
Self-referral	61/161 (38%)
Health	10/161 (6%)
Criminal justice system	8/161 (5%)
Social services	82/161 (51%)
Pre-group assessment sessions attended	1.34 (1.16) range 0–6, *n* = 137 non-missing data

### Mental health, alcohol and substance use

More than 50% had PTSD and 68% had experienced or witnessed abuse as a child (the former not necessarily due to the later). Mean PHQ-9 score was 10.88 (clinically “moderate depression”) with 18% of participants in the “moderately severe” (15–19) and “severe” (20–17) depression clinical categories. 24% reported use of non-prescription drugs and the mean hazardous drinking score was 3.99 where 4 is daily or almost daily alcohol use. 27% of the sample scored at or above threshold score of 6 for the AQ-10 (autism screening).

### Violence, motivation and risk related variables

As anticipated for a low-medium risk, voluntary DAPP, the mean ABI score for the sample was 43.61^3^ (where “no abuse” is 29 and the maximum score is 145). Eighty-four percent had children, and of those 58% had CSC and/or CAFCASS involvement.^4^ 51% were referred to the trial by Children's Social Care while 38% had self-referred. 49% percent identified as “together” or “living together” with the partner to whom they had been abusive and 63% hoped that they would be together with this partner in the future. 50% had some criminal justice involvement in the last 12 months. The mean number of pre-group assessments sessions attended was 1.34 with a range of 0–6.

### Univariate analysis

Univariate linear regression model analyses showed no association between attendance at more or fewer treatment group sessions and the following variables measured at baseline: ethnicity, having children, criminal justice involvement, experiencing or witnessing abuse as a child, ABI score, highest education achieved, type of employment, PTSD, AUDIT 3 score, AQ10 score or clinical cut-off (≥6), illicit drug use, relationship status or hope for the relationship (see Table 2).

**Table 2:** Univariate analysis.

Variable	*N*	Mean number of sessions attend (95% confidence interval)	*P*-value	Overall *p*-value for categorical variable
Pre-group assessment sessions model constant term (i.e., when pre-sessions = 0)		13.14 (10.31, 15.97)		
Pre-group assessment sessions	137	2.38 (0.78, 3.98)	0.004	
Referral source				0.0287
Self-referral (comparison group, constant term)	61	18.38 (15.57, 21.19)		
Health	10	−3.38 (−10.87, 4.11)		
Criminal justice system	8	−7.00 (−15.26, 1.25)		
Social services	82	−5.16 (−8.87, −1.45)		
Ethnicity				0.5508
White (comparison group)	139	14.66 (12.75, 16.57)		
Asian or Asian British	3	0.67 (−12.47, 13.81)		
Black/African/Caribbean/Black British	3	−0.33 (−13.47, 12.8)		
Mixed	9	6.67 (−1.08, 14.42)		
Other Ethnic Group	4	2.59 (−8.83, 14.01)		
Age model constant term (i.e., when age = 0)		7.70 (1.36, 12.03)		
Age (years)	161	0.19 (0.037, 0.35)	0.016	
No (comparison group, constant term)	71	12.59 (1.00, 8.02)		
Yes	88	4.51 (1.00, 8.02)	0.012	
Income				0.0012
Up to £5,000 (comparison group, constant term)	13	13.38 (7.35, 19.42)		
£5,000–£11,999	14	−5.81 (−14.20, 2.57)		
£12,000–£21,999	32	1.33 (−5.82, 8.49)		
£22,000–£37,999	42	6.57 (−0.34, 13.48)		
£38,000 to £72,000, and above	30	3.45 (3.78, 10.68)		
No (comparison group, constant term)	27	10.48 (6.22, 14.74)		
Yes	131	5.50 (0.83, 10.18)	0.021	
Highest education qualification achieved				0.6121
No qualifications (comparison group)	35	12.34 (8.58, 16.11)		
O-levels/GCSEs	25	4.14 (−1.70, 9.97)		
NVQ levels 1–3/GNVQ	22	2.34 (−3.72, 8.40)		
A-levels	8	6.78 (−1.95, 15.51)		
NVQ levels 4–5/HNC HND	8	4.03 (−4.70, 12.76)		
Degree/higher degree	14	2.23 (−4.81, 9.27)		
Employment				0.0905
Employed (comparison group)	88	17.10 (14.77, 19.43)		
Looking after home/family	9	−3.44 (−11.08, 4.21)		
Unemployed and looking for work	20	−5.70 (−11.12, −0.29)		
Unable to work due to long-term sickness	21	−5.82 (−11.12, −0.51)		
Retired from paid work	5	−3.50 (−13.55, 6.54)		
In full time education	1	−17.10 (−39.08, 4.87)		
Does not have children (comparison group)	24	13.79 (9.19, 18.39)		
Has children	132	1.34 (−3.66, 6.33)	0.598	
No criminal justice involvement (comparison group)	79	16.91 (14.40, 19.42)		
Self- reported criminal justice involvement	78	−3.50 (−7.06, 0.55)	0.054	
Did not experience or witness abuse as a child (comparison group)	51	15.12 (11.98, 18.25)		
Experienced or witnessed abuse as a child	107	0.097 (−3.71, 3.91)	0.960	
Self-reported CSC/CAFCASS involvement, if children<18				
No (comparison group, constant term)	56	18.38 (15.41, 21.32)		
Yes	76	−5.64 (−9.51, −1.76)	0.005	
Relationship status				0.1488
Together and living together (comparison group)	49	15.22 (12.06, 18.39)		
Together but living apart	28	−0.87 (−6.11, 4.38)		
In the process of splitting up	1	11.78 (−10.59, 34.14)		
The relationship has ended and we are living apart with no contact	28	−4.47 (−9.72, 0.77)		
The relationship has ended and we are living apart and still have contact	29	1.29 (−3.89, 6.48)		
I am not sure	17	5.07 (−1.16, 11.30)		
Something else	7	−1.08 (−10.03, 7.86)		
Relationship hopes				0.4258
That we will be together and living together (comparison group)	100	15.8 (13.56, 18.04)		
That this relationship will end	4	−5.3 (−16.72, 6.12)		
I am not sure	21	0.82 (−4.56, 6.20)		
I am in another relationship already	6	0.53 (−8.88, 9.95)		
Something else	28	−4.01 (−8.80, 0.78)		
No (comparison group)	77	15.71 (13.16, 18.27)		
Yes	82	−1.21 (−4.77, 2.34)	0.501	
AUDIT model constant term (i.e., AUDIT = 0)		15.09 (12.26, 17.91)		
AUDIT score	154	−0.032 (−0.57, 0.51)	0.908	
Drug misuse				
No (comparison group)		15.22 (13.18, 17.26)		
Yes	159	−0.57 (−4.74, 3.61)	0.790	
PHQ-9 model constant term (i.e., PHQ-9 = 0)		10.40 (7.15, 13.66)		
PHQ-9 score	159	0.43 (0.18, 0.68)	0.001	
PHQ-9 score categories				0.0369
0–4 (comparison group, constant term)	27	11.96 (7.72, 19.21)		
5–9	46	1.15 (−4.20, 6.49)		
10–14	44	3.70 (−1.69, 9.09)		
15–19	18	2.43 (−4.28, 9.14)		
20 +	20	9.64 (3.13, 16.14)		
AQ-10 autism score constant term (i.e., AQ-10 = 0)		13.39 (9.25, 17.53)		
AQ-10 score	156	0.42 (−0.44, 1.29)	0.337	
AQ-10 autism clinical cut-off < 6	114	14.88 (12.78, 16.97)		
AQ-10 autism clinical cut-off ≥6	42	1.24 (−2.79, 5.27)	0.544	
ABI model constant term (i.e., ABI = 0)		15.76 (8.04, 21.48)		
ABI	159	0.0075 (−0.14, 0.16)	0.921	
RFQ-6	156	1.34 (0.049, 2.62)	0.038	
RFQ-6 model constant term (i.e., RFQ-6 = 0)		9.09 (2.89, 15.30)		

In the univariate linear regression model analysis, for each pre-group assessment session attended there was an increase on average of 2.38 more treatment sessions attended [Table 295% confidence interval (CI): 0.78, 3.98]. For every 1 year increase in age, the average number of sessions attended increased by 0.19 (95% CI 0.037, 0.35). Being employed increased the mean number of sessions attended by 4.51 (95% CI 1.00, 8.02). The income of participants was also associated with differences in attendance, with those that were in the highest income category (£38,000 to £72,000, and above) attending 3.45 more sessions (95% CI 3.78, 10.68) than those with the lowest income (less than £5,000). However, there is no clear linear trend with increasing income increasing attendance with those in intermediate income having both having increased and decreased levels of attendance. An increased depression severity measured by the PHQ-9 was associated with an increase in the mean number of sessions attended by 0.43 (95% CI 0.18, 0.68). An increase in the RFQ-6 increases the mean number of sessions attended by 1.34 (95% CI 0.049, 2.62).

Having CSC/CAFCASS involvement was associated with reduced attendance. Men with CSC/CAFCASS attended on average 5.64 fewer sessions than those without (95% CI −9.51, −1.76).

An interesting result is that those men who disclosed their income (*n* = 131) attended an average of 5.50 more sessions (95% CI 0.83, 10.18) compared with than those who did not disclose their income (*n* = 27).

### Multivariable analyses

Multivariable linear regression model analysis indicated that higher baseline depression symptoms (PHQ-9), income, and greater pre-assessment engagement were associated with increased programme attendance, explaining approximately 18% of the variance (Table 3 adjusted *R*2 value = 0.1813, *n* = 134). The covariate age was also included since age may confound the association between income and number of sessions attended. A dummy variable for not reporting income was also included, where if income was not reported, values of income were coded as the lowest income category. The addition of other variables potentially associated with programme attendance (employment status, reflective functioning (RFQ-6), referral source, CSC/CAFCASS) involvement did not improve model fit in multivariable analysis. Interaction effects between covariates were not tested—as such, potential synergistic relationships between these variables are unknown. The residuals of this model were plotted, and there was limited evidence of departure from normality.

**Table 3:** Multivariable analysis.

Variable	Mean number of sessions attend (95% confidence interval)	*P*-value
Constant term	5.06 (−4.26, 14.38)	
PHQ-9 score	0.39 (0.13, 0.65)	0.003
Age	0.07 (−0.096, 0.25)	0.380
Income report		
Yes	Comparison group	
No	−3.54 (−11.28, 4.20)	0.367
Income		
£5,000–£11,999	−4.57 (−13.71, 4.57)	0.057 (overall)
£12,000–£21,999	1.19 (−6.31, 8.70)	
£22,000–£37,999	5.67 (−1.62, 12.95)	
£38,000 to £72,000, and above	3.75 (−4.01, 11.50)	
Number of pre-assessment sessions attended	1.70 (0.13, 3.26)	0.034

### Exploratory analyses

In univariate logistic regression model analyses there is evidence of an association between RFQ-6 and reporting income (outcome), with those having a higher RFQ-6 score, having higher odds of reporting income (Odds ratio, OR, 1.39, 95% CI 1.03, 1.88). There is no association between criminal justice involvement and income reporting (OR 0.49, 95% CI 0.20, 1.19). There is no association between RFQ-6 and criminal justice involvement (not reported in table OR −0.10, 95% CI −0.33, 0.13; Table 4).

**Table 4:** Univariate analyses of reporting income.

Variable	Income report (yes): Odds ratio (95% confidence in interval)	*P*-value
RFQ-6 model constant term (i.e., RFQ-6 = 0)	1.16 (0.31, 4.38)	
RFQ-6	1.39 (1.03, 1.88)	0.031
Criminal justice involvement		
No (comparison group, constant term)	7.78 (3.89, 15.57)	
Yes	0.49 (0.20, 1.19)	0.115

In univariate logistic regression model analyses there is no evidence of an association between RFQ-6 and CSC/CAFCASS involvement, PHQ-9, drug misuse, alcohol misuse, ABI or PTSD and CSC/CAFCASS involvement.

In a univariate linear regression analysis of number of group sessions attended on RFQ-6 and pre-group sessions attended there was evidence that, after adjustment for RFQ-6, for each pre-group session attended there was a mean increase of 2.28 group sessions attended (95% CI 0.64, 3.92, *p*-value = 0.007).

## Discussion

This research highlights four distinct areas of that were relevant to attendance on the REPROVIDE trial: (1) Life stability—including age, employment and income; (2) Trust in professionals—including pre-group session, reflective functioning and disclosure of income; (3) Depression severity and; (4) Self-reported Children's Social Care/Child and Family Court Advisory Service involvement. We address each of these in turn below.

### A more stable life? Age, employment and income

Consistent with previous research on DAPP attendance, older age (Askeland and Heir, 2013; Cunha et al., 2023; Lauch et al., 2017; Priester et al., 2019; Scott et al., 2013) being employed (Lauch et al., 2017; Rooney and Hanson, 2001; Scott et al., 2011, 2013) and having a higher income (Brodeur et al., 2008; Buttell and Carney, 2008; Rondeau et al., 2001) was associated with increased attendance in univariate analysis. These demographic features may position participants at a point in life where the disbenefits of abuse begin to outweigh the benefits (Hester et al., 2006) and/or with sufficient life-stability due to employment and greater income such that attending a behavior change programme becomes more manageable. Interestingly, while overall, employment was associated with attendance, there was no linear trend in attendance by income. Attendance was highest among the lowest-income group ( ≤ £5,000), then dropped sharply in the second-lowest group (£5,000–£11,999), before rising again in the middle-income brackets. This may reflect differences in time-availability and employment flexibility: the lowest-income group may include individuals not in work, while the second-lowest group may be more likely to be in insecure or inflexible employment. Attendance remained higher in the middle-income groups, possibly due to greater job stability and within-job-autonomy, with a slight decline in the highest-income group. In multivariable analyses, a weak association with income remained present but neither age nor employment were associated with attendance. More strongly associated with attendance than income, was whether or not participants disclosed income at all.

### Trust issues? Pre-group sessions, reflective functioning and disclosure of income

In the univariate analyses, attending pre-group sessions, having higher RFQ-6 score and disclosure of income were all correlated with increased session attendance.

Though no prior studies have demonstrated a correlation between reflective functioning and DAPP, attendance, RF has been shown to predict attendance in Mentalisation-Based Group Therapy (MBG-T; Hauschild et al., 2024; Jørgensen et al., 2021). Meanwhile the related concepts of emotional decoding and cognitive empathy abilities have been associated with higher dropout and risk of recidivism (Romero-Martínez et al., 2023a). *Reflective functioning* is synonymous with *mentalising* which

“refers to the capacity to reflect on internal mental states such as feelings, wishes, goals, and attitudes, with regard to both the self and others” (Fonagy et al., 2016, p. 2)

This kind of self-reflection is central to the REPROVIDE intervention which seeks to change the participants' relationships with others by eliciting empathy and cultivating recognition of the impacts on others of own behavior. It follows then that in a group within which mentalising is a central feature, a participant's baseline ability to mentalise may predict attendance.

Multivariable analysis, including pre-group sessions attended, reflective functioning and disclosure of income, showed that disclosure of income and pre-group session attendance were associated with number of group sessions attended (Table 3). The following hypotheses may help explain these associations:

#### Non-disclosure of income

Firstly, it was hypothesized that non-disclosure of income may be linked to participants' criminality—if a proportion of their income was derived from criminal activity, they may not have wished to disclose income. Multivariable analysis showed no evidence of an association between criminal justice involvement in the last 12 months and increased odds of reporting income. Unlike studies of court-mandated DA perpetrators, neither was there an association between criminal justice involvement and attendance in the univariate analysis.

Secondly, it was hypothesized that non-disclosure of income may have to do with a lack of trust in professionals/researchers or poor working alliance. Working or Therapeutic Alliance is composed of three components—(1) client-therapist (in this case, facilitator-group member) agreement on the goals of therapy, client-therapist agreement on the tasks required and (3) the client-therapist “bond”—a key feature of which is trust (Flückiger et al., 2018). In a meta-analysis of 190 mental health and substance misuse studies covering more than 14,000 treatments, (Horvath et al. 2011) found a consistent positive effect (*r* = 0.275) of working alliance (WA) on treatment outcome and numerous studies in this field have also shown association between WA and treatment compliance. In DA research, observer-reported WA in the first few sessions has been was associated with significant reductions of physical and psychological violence at the end of the intervention (Brown and O'Leary, 2000). (Taft et al. 2003) found that therapist-reported WA was associated with significant reductions of physical and psychological violence at 6-month follow-up while (Santirso et al., 2020b) found WA to be associated with increased engagement and attendance in a court-mandated DAPP.

In our study we did not have a measure of trust in professionals or a *direct* measure of working alliance [for a sub-study exploring WA within the REPROVIDE intervention see: Fowler et al., 2021]. Moreover, given that the disclosure of income question was asked in the baseline questionnaire, there was little practitioner-participant contact time to build a working alliance prior to this question being asked. However, reflective functioning (RFQ-6) may predict or offer proxy for “ability to build working alliance”. In a small study (*n* = 24), (Müller et al. 2006) found that RF predicted therapy success and showed a positive but not statistically significant (*p* = 0.082, ES = 0.95) correlation between RF and working alliance. Meanwhile, (Ekeblad et al. 2016) using the DSRF—an RF scale modified for patients with Major Depressive Disorder—also found that lower RF predicted lower patient and therapist-rated WA across treatment. It is conceivable then that higher reflective functioning may position participants as more trusting of professionals at baseline, prior to any trust-building activities having taken place. Supporting the idea that willingness to disclose income may have to do with trust in professionals, and that this in turn may predict group attendance, univariate analysis revealed an association between higher reflective functioning and higher odds of reporting income.

#### Pre-group session attendance

Pre-group session attendance was also associated with group attendance. There may be a motivational dimension here. While not a strictly practiced motivational interviewing (MI) intervention, MI is central to DAPP practice in the UK (Bates et al., 2017; Fowler et al., 2021) and to [TRIAL NAME]. The REPROVIDE facilitator training and manual are deeply MI-informed, and facilitators were trained to use techniques commonly used in MI (including goal setting, developing discrepancy, reframing, and encouraging self-efficacy). Motivational strategies integrated with DAPPs have been shown to increase pro-social behaviors and working alliance (Santirso et al., 2020b), as well as to increase treatment adherence and reduce recidivism (Lila et al., 2025).

On REPROVIDE, pre-group sessions were typically conducted one-to-one and offered a space to explore emotional (e.g., fear of group settings) and practical needs (e.g., assistance with transport or accessibility), as well as to set goals and tasks in advance of the group starting. By helping participants to feel that their needs are being met and using MI techniques, these sessions may help build trust and working alliance which better equips participants to attend the group for more sessions than those who participate in no or fewer pre-group sessions.

An alternative explanation is simply that participants who are more likely attend pre-group sessions are more likely to attend group sessions—so the content of the pre-group sessions are immaterial. Were this the case we might expect to see RF “driving” attendance in multivariable analysis—there was no evidence of this. What may be happening is that on average, those with higher RF find it easier to attend a pre-group session and bond with a facilitator but it is additional work that goes on within this pre-group session (building WA through MI) which makes them more likely to attend a higher number of sessions.

### Depression severity

Men with high levels of depression severity are typically overrepresented in DAPPs, and suicidality and high harm domestic abuse perpetration are known to intersect (Knipe et al., 2024). REPROVIDE participants showed high levels of depression severity and suicidality with over half (52%) scoring above the clinical threshold for moderate depression and 13% (*n* = 20) scoring above the threshold for severe depression (triggering safeguarding actions). Given the existing evidence on the correlation between other psychopathologies and DAPP attrition (e.g., Munro and Sellbom, 2022; Romero-Martínez et al., 2021, 2023b), we might anticipate that depression severity would predict reduced attendance. However, increased depression severity measured by the PHQ-9 was associated with an increase in attendance in a multivariable analysis. Previous studies of non-DA behavior change group-based interventions have shown associations between depression severity and attendance—positively in mental health, eating disorder and substance use treatments (Gibbons et al., 2024) and negatively in weight loss treatment (Shell et al., 2020). While no other studies of group-based DAPPs have identified an association between depression severity and attendance, research on the Drive programme—a one-to-one intervention for high-risk domestic abuse perpetrators—similarly found high levels of mental health need among “high-engaging” participants. This was attributed in part to limited mental health provision within the wider multi-agency ecosystem (Hester et al., 2019, 2025). REPROVIDE operated within the same context of scarce mental health services, meaning that individuals with elevated needs may have had few alternative sources of support. Participants may have additionally derived perceived mental health benefits from the peer-supportive group setting and facilitator engagement (Cramer et al., 2024). The presence of this association suggests—consistent with findings elsewhere (Hester et al., 2006)—that participants may be more likely to attend when they perceive personal benefit in doing so.

### Motivation or complexity—children's social care/CAFCASS involvement

The only factor associated with decreased attendance was self-reported Children's Social Care (CSC) or Children and Family Court Advisory and Support Service (CAFCASS) involvement at baseline.^5^ The simplest explanation for reduced attendance among this subgroup is that this is a more complex, higher-risk and/or “harder-to-engage” group. According to this hypothesis, risk/life-instability drives reduced attendance, and this is the reason CSC are involved rather than CSC-involvement driving reduced attendance. However, were this explanation true, we would expect to see a signal in the violence or risk-related variables—for example higher ABI scores, substance or alcohol use, CJ involvement, PTSD prevalence or Phq-9 scores. Multivariable analysis detected no such signal.

The role of CSC involvement as a predictor of attrition is under-researched. Contra to our findings here, a small study by (Stanley et al. 2012) found that voluntary DAPP men who were involved with CSC were *more likely* than other programme participants to complete more than five sessions. This, they argued, was because while CSC involvement initially produced a form of *extrinsic motivation* where men felt compelled to attend due to threat of care proceedings. As the group progressed, this extrinsic motivation transforms into *intrinsic motivation* as men begin to see the benefits becoming “better fathers” both for themselves and for their children. The evaluation of the Drive Pilot (Hester et al., 2019) similarly found that “high-engaging” men attended due to combination of compulsion (e.g., criminal justice sanctions or care proceedings) and voluntarism whereby the former gave way to the latter as men built trust in professionals and saw the benefits of engaging—or felt *intrinsically motivated*. This aligns with the motivational interviewing approach used in REPROVIDE (and Drive) which seeks to develop internal or intrinsic motivations in the client.

A third possibility accords with the initial simple explanation that the CSC-involved sub-group are a higher-risk and/or “harder to engage” group but that this does not show up in their (self-reported) risk-related variables due to a lack of trust in professionals. Though not documented with perpetrators, CSC-involved parents have been shown to under-disclose risk due to fear of escalating care proceedings (Bacon et al., 2023). Given the heightened scrutiny of families where DA is present, REPROVIDE may similarly see under-reporting of other risk factors where CSC are involved. Interestingly, there is a trend, although not statistically, that men with higher reflective functioning (scoring higher on the RFQ-6) were less likely to have CSC involved (Table 5), and as argued above, low RF may be linked to lack of trust in professionals—which would suggest underreporting of risk. Furthermore, we know that CSC/CAFCASS men are overrepresented in the 0 sessions attended group (28% of 76 compared with 14% of 56)—this challenges the theory that they are initially *extrinsically* motivated and then cease to participate later. Or it suggests that if they are extrinsically motivated, they are not any more motivated by this than non-CSC/CAFCASS men are. This then suggests they may simply be more difficult to engage.

**Table 5:** Univariate analysis of CSC/CAFCASS involvement.

Variable	CSC/CAFCASS involvement (yes): Odds ratio (95% confidence in interval)	*P*-value
PHQ-9 model constant term (i.e., PHQ-9 = 0)	1.74 (0.92, 3.31)	
PHQ-9	0.98 (0.93, 1.03)	0.363
RFQ-6 model constant term (i.e., RFQ-6 = 0)	4.29 (1.24, 14.86)	
RFQ-6	0.78 (0.60, 1.00)	0.057
ABI model constant term (i.e., ABI = 0)	3.28 (0.88, 12.17)	
ABI	0.98 (0.95, 1.00)	0.172
Illicit drug use: no (comparison group, constant term)	1.43 (0.96, 2.12)	
Yes	0.80 (0.35, 1.81)	0.593
AUDIT model constant term (i.e., AUDIT = 0)	1.73 (0.99, 3.03)	
AUDIT score	0.93 (0.84, 1.04)	0.199
PTSD		
No (comparison group)	1.46 (0.89, 2.41)	
Yes	0.87 (0.43, 1.73)	0.685

## Strengths and limitations

A strength of this study is that the dependent variable (attendance) was derived from administrative records rather than self-report, reducing uncertainty about whether sessions were attended.

The study also has several limitations. The modest sample size, which is made smaller by missing attendance data, limits statistical power. This is reflected in the wide confidence intervals around several association estimates. Some explanatory variables relied on self-report and may be under-reported (this is less likely than overreporting for this cohort). In addition to this, attrition may have been influenced by factors which were beyond the scope of this research—for example, distance traveled and transport connectivity, particularly in more rural areas. Additionally, attendance of the CSC-involved group, may also be affected by local relationships between DAPP providers and CSC teams—for example, how much information sharing and collaboration exists and to what extent this works to hold men to account or extrinsically motivate them to continue.

## Conclusion

In a field in which most studies explore attrition in the context of court-mandated DAPPs, this research provides a critical insight into the factors correlated with attendance in the most common form of DAPP in the UK—voluntary programmes. Attrition is costly to services, diverts resources from other participants who are already engaged and fails to address the risk that DAPPs aim to reduce or eliminate: DA perpetration. Our findings point to a set of implications for practice and further research. Given the association between pre-group sessions and attendance, this research underlines the importance of trust building and attending to individual needs prior to group intake. As has been found elsewhere (Lila et al., 2025), pre-sessions using motivational interviewing (MI) strategies may enhance attendance. Given the links between reflective functioning (RF), trust in professionals and working alliance, RF screening and optional income disclosure may help practitioners to see where additional resource will be required to establish/build trust such that the participant feels able to attend a greater number of sessions. Counter to previous research on a voluntary DAPP (Stanley et al., 2012), our findings on CSC-involved men suggest that additional effort is required, perhaps including enhanced multi-agency collaboration, to enable these men to stay engaged in a voluntary DAPP—this is especially important given the presence of children and known risk in these cases. That men with high levels of suicidality are overrepresented among DA perpetrators is well established (Knipe et al., 2024) but that this group may be more likely to attend a higher number of sessions is an important result and highlights the importance of facilitator training and skill in the identification and risk management around men's mental health and the ways in which DAPPs may serve a dual function across health and crime prevention. Equally, and while it is encouraging that these men tend to stay engaged this may reflect a paucity of mental health provision more widely, and practitioner focus here consumes considerable time which might be more effectively oriented to their specialism—DA behavior change. Conversely, if adequately resourced, rather than see depression severity as a risk to attendance at screening, it may be understood as a lever or incentive for engagement.

## Data Availability

The datasets presented in this article are not readily available due to the highly sensitive nature of the data which might place victim survivors of domestic abuse at further risk. Data requests will require a formal proposal and will be reviewed by the University of Bristol Data Access Committee. A signed data access agreement will also be required (Please refer to the following link on how to access data held in the University of Bristol's Research Data repository: https://www.bristol.ac.uk/staff/researchers/data/accessing-research-data/). The quantitative datasets generated and analysed during the study will be made available by the authors upon reasonable request.

## Footnotes

1 See Appendix A for a summary of the intervention in table form.

2 This is difficult to compare to national statistics which is done in percentiles, the 10^th^ Percentile for 23/24 earned 10,800 which suggests the sample may have a higher proportion of low-earners in comparison to national averages.

3 The ABI 29 is scored using a 5-point Likert scale with a minimum total score of 29 and a maximum of 145 where 29 is no abuse reported and 145 is all behaviors listed at the highest frequency possible to report. There are no agreed clinical cutoffs for the ABI-29.

4 Children's Social Care (CSC) or CAFCASS involvement is self-reported and means that either or both of these services were involved in the man's family/life. This is separate from whether or not the man was referred by these services—for example, CSC may have been involved with the family but the man self-referred. Since CAFCASS involvement was an exclusion criterion, men referred by CAFCASS were not included. Due to the way in which the baseline questions were asked, it was not possible to differentiate between self-reported CSC and CAFCASS involvement.

5 Self-reported CSC/CAFCAS involvement was collected using the IMPACT toolkit (Vall et al., 2021) which due to the way the questions are phrased does not allow the disaggregation of CSC vs. CAFACSS involvement. However, having an open CAFCASS case was an exclusion criterion for participation so this measure will overwhelmingly capture CSC involvement.

## Appendix A

**Table A1:** Intervention description table.

Intervention	Eligibility	Intervention core features
REPROVIDE men's behavior change programme	Men Over 21 years of age Admit to some form of abusive behavior towards a female intimate partner/ex-partner. Not court-mandated to attend. Not involved in ongoing criminal justice proceedings. Not involved in ongoing child and family court proceeding.	Duration: 23 weeks + 6 optional relapse prevention sessions offered monthly Group based: Yes Manualised: Yes Modality: Hybrid (Duluth + CBT + Trauma informed approaches + Motivational Interviewing) Facilitation: Two facilitators, mixed genders Integrated support for ex-/partners: Yes

## References

[B1] Arce R. Arias E. Novo M. Fariña F. (2020). Are interventions with batterers effective? A meta-analytical review. Psychosoc. Interven. 29:11. doi: 10.5093/pi2020a11

[B2] Askeland I. R. Heir T. (2013). Early dropout in men voluntarily undergoing treatment for intimate partner violence in Norway. Violence Vict. 28, 822–831. doi: 10.1891/0886-6708.VV-D-12-00137 24364125

[B3] Babcock J. C. Gallagher M. W. Richardson A. Godfrey D. A. Reeves V. E. D'Souza J. (2024). Which battering interventions work? An updated meta-analytic review of intimate partner violence treatment outcome research. Clin. Psychol. Rev. 111:102437. doi: 10.1016/j.cpr.2024.102437 38810357

[B4] Bacon G. Sweeney A. Batchelor R. Grant C. Mantovani N. Peter S. . (2023). At the edge of care: a systematic review and thematic synthesis of parent and practitioner views and experiences of support. Health Soc. Care Comm. 2023:6953134. doi: 10.1155/2023/6953134

[B5] Bates E. A. Graham-Kevan N. Bolam L. T. Thornton A. J. V. (2017). A review of domestic violence perpetrator programmes in the United Kingdom. Partner Abuse 8, 3–46. doi: 10.1891/1946-6560.8.1.3

[B6] Beldin K. L. (2008). Social information processing, program completion, and recidivism: One court's referrals to a batterer intervention program (Master's thesis, Case Western Reserve University). OhioLINK. Available online at: http://rave.ohiolink.edu/etdc/view?acc_num=case1207177110 (Accessed November 17, 2025).

[B7] Bell C. Coates D. (2022). The effectiveness of interventions for perpetrators of domestic and family violence: an overview of findings from reviews. ANROWS. Available online at: https://www.anrows.org.au (Accessed November 17, 2025).

[B8] Bennett L. W. Hsieh C.-M. Stoops C. (2010). Underclass men in batterer intervention programs: Disorders and disparities. Fam. Soc. 91, 394–400. doi: 10.1606/1044-3894.4038

[B9] Bowen E. Gilchrist E. (2004). Do court- and self-referred domestic violence offenders share the same characteristics? Legal Criminol. Psychol. 9, 279–294. doi: 10.1348/1355325041719383

[B10] Bowen E. Gilchrist E. (2006). Predicting dropout of court-mandated treatment in a British sample of domestic violence offenders. Psychol. Crime Law 12, 573–587. doi: 10.1080/10683160500337659

[B11] Brodeur N. Rondeau G. Brochu S. Lindsay J. Phelps J. (2008). Does the transtheoretical model predict attrition in domestic violence treatment programs? Viol. Vict. 23, 493–507. doi: 10.1891/0886-6708.23.4.493 18788340

[B12] Brown P. D. O'Leary K. D. (2000). Therapeutic alliance: predicting continuance and success in group treatment for spouse abuse. J. Consult. Clin. Psychol. 68, 340–345. doi: 10.1037/0022-006X.68.2.340 10780135

[B13] Buttell F. P. Carney M. M. (2002). Psychological and demographic predictors of attrition among batterers court ordered into treatment. Soc. Work Res. 26, 31–41. doi: 10.1093/swr/26.1.31

[B14] Buttell F. P. Carney M. M. (2008). Batterer intervention program attrition: evaluating the impact of state program standards. Res. Soc. Work Pract. 18, 177–188. doi: 10.1177/1049731508314277

[B15] Buttell F. P. Pike C. K. (2002). Investigating predictors of treatment attrition among court-ordered batterers. J. Soc. Serv. Res. 28, 53–68. doi: 10.1300/J079v28n04_03

[B16] Cantos A. L. Goldstein D. A. Brenner L. O'Leary K. D. Verborg R. (2015). Correlates and program completion of family-only and generally violent perpetrators of intimate partner violence. Behav. Psychol. Psicol. Conduct. 23, 549–569.

[B17] Carney M. M. Buttell F. P. Muldoon J. (2006). Predictors of batterer intervention program attrition. J. Offender Rehabil. 43, 35–54. doi: 10.1300/J076v43n02_02

[B18] Chang H. Saunders D. G. (2002). Predictors of attrition in two group programs for men who batter. J. Fam. Violence 17, 273–292. doi: 10.1023/A:1016057328929

[B19] Cissner A. B. Puffett N. K. (2006). Do Batterer Program Length or Approach Affect Completion or Re-Arrest Rates? Center for Court Innovation.

[B20] Cramer H. Gaunt D. M. Shallcross R. Bates L. Kandiyali R. Sardinha L. M. . (2024). Randomised pilot and feasibility trial of a group intervention for men who perpetrate intimate partner violence against women. BMC Public Health 24:18640. doi: 10.1186/s12889-024-18640-5 PMC11055266 38678198

[B21] Cuevas D. A. Bui N. H. (2016). Social factors affecting the completion of a batterer intervention program. J. Fam. Violence 31, 95–107. doi: 10.1007/s10896-015-9748-0

[B22] Cunha O. Pedrosa J. Silva Pereira B. Caridade S. de Castro Rodrigues A. Braga T. (2024). Intervention program dropout among perpetrators of intimate partner violence: a meta-analysis. Trauma Violence Abuse 25, 2735–2751. doi: 10.1177/15248380231224036 38323403

[B23] Cunha O. Silva A. Cruz A. R. de Castro Rodrigues A. Braga T. Gonçalves R. A. (2023). Dropout among perpetrators of intimate partner violence attending an intervention program. Psychol. Crime Law 29, 634–652. doi: 10.1080/1068316X.2022.2030337

[B24] Daly J. E. Pelowski S. (2000). Predictors of dropout among men who batter. Violence Vict. 15, 137–160. doi: 10.1891/0886-6708.15.2.137 11108498

[B25] Daly J. E. Power T. G. Gondolf E. W. (2001). Predictors of batterer program attendance. J. Interpers. Violence 16, 971–991. doi: 10.1177/088626001016010001

[B26] DeHart D. D. Kennerly R. J. Burke L. K. Follingstad D. R. (1999). Predictors of attrition in a treatment program for battering men. J. Fam. Violence 14, 19–34. doi: 10.1023/A:1022861809014

[B27] Duplantis A. D. Romans J. S. C. Bear T. M. (2006). Persistence in domestic violence treatment. J. Aggres. Maltreat. Trauma 13, 1–18. doi: 10.1300/J146v13n01_01

[B28] Eckhardt C. Holtzworth-Munroe A. Norlander B. Sibley A. Cahill M. (2008). Readiness to change, partner violence subtypes, and treatment outcomes. Violence Vict. 23, 446–475. doi: 10.1891/0886-6708.23.4.446 18788338

[B29] Eckhardt C. I. Samper R. E. Murphy C. M. (2008). Anger disturbances among perpetrators of intimate partner violence. J. Interpers. Violence 23, 1600–1617. doi: 10.1177/0886260508314322 18378815

[B30] Ekeblad A. Falkenström F. Holmqvist R. (2016). Reflective functioning as predictor of working alliance and outcome. J. Consult. Clin. Psychol. 84, 67–78. doi: 10.1037/ccp0000055 26594944

[B31] Expósito-Álvarez C. Roldán-Pardo M. Vargas V. Maeda M. Lila M. (2025). The impact of trauma and substance use on emotion regulation and intimate partner violence perpetration. Behav. Sci. 15:156. doi: 10.3390/bs15020156 40001789 PMC11852141

[B32] Fitz-Gibbon K. Helps N. Ralph B. (2024). Engaging in Change: A Victorian Study of Perpetrator Program Attrition and Engagement. Melbourne: Monash University.

[B33] Flückiger C. Del Re A. C. Wampold B. E. Horvath A. O. (2018). The alliance in adult psychotherapy: a meta-analytic synthesis. Psychotherapy 55, 316–340. doi: 10.1037/pst0000172 29792475

[B34] Fonagy P. Luyten P. Moulton-Perkins A. Lee Y.-W. Warren F. Howard S. Ghinai R. Fearon P. Lowyck B. (2016). Development and validation of a self-report measure of mentalizing. PLOS ONE 11:e0158678. doi: 10.1371/journal.pone.0158678 27392018 PMC4938585

[B35] Fowler J. Morgan K. Eisenstadt N. Feder G. Cramer H. (2021). An exploration of the working alliance in group programmes for domestic abuse perpetrators. Reconnect. Available online at: https://reconnect.org.uk/wp-content/uploads/2024/12/Fowler-etal-2021-An-exploration.pdf (Accessed November 17, 2025).

[B36] Gerlock A. A. (2001). A profile of who completes and who drops out of domestic violence rehabilitation. Issues Ment. Health Nurs. 22, 379–400. doi: 10.1080/01612840151136911 11885155

[B37] Gibbons J. B. Cox S. A. Straub L. Au J. S. Wang P. S. Liu J. . (2024). Association between depression severity, recovery, and dropout from behavioral health care. Psychiatry Res. Commun. 4:100185. doi: 10.1016/j.psycom.2024.100185 PMC13574114 42741755

[B38] Hauschild S. Dragovic D. Kasper L. Sobanski E. Taubner S. (2024). Patient characteristics of completion and dropout of mentalization-based treatment. Front. Psychol. 15:1390169. doi: 10.3389/fpsyg.2024.1390169 39417025 PMC11480065

[B39] Hester M. Eisenstadt N. Ortega-Avila A. G. Morgan K. J. Walker S.-J. Bell J. (2019). Evaluation of the Drive Project. Bristol, UK: University of Bristol. Available online at: https://drivepartnership.org.uk/publication/university-of-bristol-evaluation-of-the-drive-project/ (Accessed November 17, 2025).

[B40] Hester M. Ortega-Avila A. G. Eisenstadt N. Walker S.-J. (2025). Evaluation of the Drive intervention using a quasi-experimental approach. Soc. Sci. 14:55. doi: 10.3390/socsci14020055

[B41] Hester M. Westmarland N. Gangoli G. Wilkinson M. O'Kelly C. Kent A. . (2006). Domestic Violence Perpetrators: Identifying Needs to Inform Early Intervention. Bristol: University of Bristol in association with the Northern Rock Foundation and the Home Office. doi: 10.1080/09627250608553400

[B42] Horvath A. O. Del Re A. C. Flückiger C. Symonds D. (2011). Alliance in individual psychotherapy. Psychotherapy 48, 9–16. doi: 10.1037/a0022186 21401269

[B43] Jewell L. M. Wormith J. S. (2010). Variables associated with attrition from domestic violence treatment programs. Crim. Justice Behav. 37, 1086–1113. doi: 10.1177/0093854810376815

[B44] Johnston I. Robinson A.L. Dahlby L. Smith E. Kumar S.M. Gilchrist E. . (2026). Methodological challenges in determining the effectiveness of intimate partner violence perpetrator programmes: a systematic review. Trauma Violence Abus. 1–16. doi: 10.1177/15248380261437078 42011535

[B45] Jørgensen M. S. Bo S. Vestergaard M. Storebø O. J. Sharp C. Simonsen E. (2021). Predictors of dropout from mentalization-based group treatment. Psychother. Res. 31, 950–961. doi: 10.1080/10503307.2020.1871525 33428543

[B46] Knipe D. Vallis E. Kendall L. Snow M. Kirkpatrick K. Jarvis R. . (2024). Suicide rates in high-risk domestic abuse perpetrators. Crisis 45, 242–245. doi: 10.1027/0227-5910/a000925 37606346

[B47] Lauch K. M. R. Hart K. J. Bresler S. (2017). Predictors of treatment completion and recidivism among IPV offenders. J. Aggres. Maltreat. Trauma 26, 543–557. doi: 10.1080/10926771.2017.1299824

[B48] Lila M. Expósito-Álvarez C. Roldán-Pardo M. (2025). Motivational strategies reduce recidivism and enhance adherence. Front. Psychiatry 16:1538050. doi: 10.3389/fpsyt.2025.1538050 39950177 PMC11821649

[B49] Lila M. Gracia E. Catalá-Miñana A. (2020). More likely to dropout, but what if they don't? J. Interpers. Violence 35, 1958–1981. doi: 10.1177/0886260517699952 29294698

[B50] Lila M. Martin-Fernandez M. Gracia E. Lopez-Ossorio J. J. Gonzalez J. L. (2019). Identifying predictors of recidivism. Psychosoc. Interven. 28, 157–167. doi: 10.5093/pi2019a19

[B51] Lilley-Walker S.-J. Hester M. Turner W. (2018). Evaluation of European domestic violence perpetrator programmes. Int. J. Offender Ther. Comp. Criminol. 62, 868–884. doi: 10.1177/0306624X16673853 27884945

[B52] Morgan K. Man M.-S. Bloomer R. Cochrane M. Cole M. Dheensa S. . (2023). Effectiveness and cost-effectiveness of a perpetrator programme. Trials 24:761. doi: 10.1186/s13063-023-07612-6 37770906 PMC10540403

[B53] Müller C. Kaufhold J. Overbeck G. Grabhorn R. (2006). The importance of reflective functioning. Psychol. Psychother. 79, 485–494. doi: 10.1348/147608305X68048 17312866

[B54] Munro O. E. Sellbom M. (2022). Borderline personality traits in IPV intervention. Psychol. Crime Law 28, 489–510. doi: 10.1080/1068316X.2021.1929976

[B55] Nesset M. B. Lara-Cabrera M. L. Dalsbø T. K. Pedersen S. A. Bjørngaard J. H. Palmstierna T. (2019). Cognitive behavioral group therapy for male perpetrators. BMC Psychiatry 19:11. doi: 10.1186/s12888-019-2010-1 30621661 PMC6325780

[B56] Office for National Statistics (2021). Education, England and Wales: Census 2021. Available online at: https://www.ons.gov.uk (Accessed November 17, 2025).

[B57] Office for National Statistics (2024). Employment in the UK: April 2024. Available online at: https://www.ons.gov.uk (Accessed November 17, 2025).

[B58] Priester M. A. Kulkarni S. Mennicke A. Bell B. A. (2019). Factors associated with batterer intervention program attrition. Violence Vict. 34, 296–311. doi: 10.1891/0886-6708.VV-D-17-00067 31019013

[B59] Richards T. N. Jennings W. G. Murphy C. (2021). Risk and protective factors for program attrition. J. Interpers. Violence 36, NP8464–NP8487. 10.1177/0886260519834096 30852952

[B60] Rock R. C. Sellbom M. Ben-Porath Y. S. Salekin R. T. (2013). Validity of psychopathy in a batterers' intervention sample. Law Hum. Behav. 37, 145–154. doi: 10.1037/lhb0000006 22746285

[B61] Romero-Martínez Á. Lila M. Gracia E. Martín-Fernández M. Moya-Albiol L. (2021). Antisocial batterers, compliance, and recidivism. Psychol. Violence 11, 318–328. doi: 10.1037/vio0000296

[B62] Romero-Martínez Á. Lila M. Sarrate-Costa C. Comes-Fayos J. Moya-Albiol L. (2023a). Dropout and recidivism explained by socio-cognitive deficits. Aggress. Behav. 49, 222–235. doi: 10.1002/ab.22064 36449417

[B63] Romero-Martínez Á. Lila M. Sarrate-Costa C. Comes-Fayos J. Moya-Albiol L. (2023b). ADHD, neuropsychological deficits, and treatment dropout. Eur. J. Psychol. Appl. Legal Context 15, 33–42. doi: 10.5093/ejpalc2023a4

[B64] Rondeau G. Brodeur N. Brochu S. Lemire G. (2001). Dropout and completion of treatment among spouse abusers. Violence Vict. 16, 127–143. doi: 10.1891/0886-6708.16.2.127 11345474

[B65] Rooney J. Hanson R. K. (2001). Predicting attrition from treatment programs. J. Fam. Violence 16, 131–149. doi: 10.1023/A:1011106902465

[B66] Santirso F. A. Gilchrist G. Lila M. Gracia E. (2020a). Motivational strategies in IPV interventions. Psychosoc. Interven. 29, 175–190. doi: 10.5093/pi2020a13

[B67] Santirso F. A. Lila M. Gracia E. (2020b). Motivational strategies, alliance, and protherapeutic behaviors. Eur. J. Psychol. Appl. Legal Context 12, 77–84. doi: 10.5093/ejpalc2020a7

[B68] Scott K. King C. McGinn H. Hosseini N. (2011). Effects of motivational enhancement. J. Fam. Violence 26, 139–149. doi: 10.1007/s10896-010-9353-1

[B69] Scott K. King C. McGinn H. Hosseini N. (2013). The (dubious?) benefits of second chances. J. Interpers. Violence 28, 1657–1671. doi: 10.1177/0886260512468321 23277469

[B70] Scott K. L. (2004). Stage of change as a predictor of attrition. J. Fam. Violence 19, 37–47. doi: 10.1023/B:JOFV.0000011581.01231.1e

[B71] Shell A. L. Hsueh L. Vrany E. A. Clark D. O. Keith N. C. R. Xu H. Stewart J. C. (2020). Depressive symptom severity and attendance. J. Psychosom. Res. 131:109970. doi: 10.1016/j.jpsychores.2020.109970 32088427 PMC7429242

[B72] Stanley N. Graham-Kevan N. Borthwick R. (2012). Fathers and domestic violence. Child Abuse Rev. 21, 264–274. doi: 10.1002/car.2222

[B73] Stoops C. Bennett L. Vincent N. (2010). Behavior-based typology of men who batter. J. Fam. Violence 25, 325–335. doi: 10.1007/s10896-009-9294-8

[B74] Taft C. T. Murphy C. M. Elliott J. D. Keaser M. C. (2001a). Race and demographic factors in treatment attendance. J. Fam. Violence 16, 385–400. doi: 10.1023/A:1012224910252

[B75] Taft C. T. Murphy C. M. Elliott J. D. Morrel T. M. (2001b). Attendance-enhancing procedures in group counseling. J. Couns. Psychol. 48, 51–60. doi: 10.1037/0022-0167.48.1.51

[B76] Taft C. T. Murphy C. M. King D. W. Musser P. H. Dedeyn J. M. (2003). Process and treatment adherence factors in CBT. J. Consult. Clin. Psychol. 71, 812–820. doi: 10.1037/0022-006X.71.4.812 12924686

[B77] Travers Á. McDonagh T. Cunningham T. Armour C. Hansen M. (2021). Effectiveness of interventions to prevent recidivism. Clin. Psychol. Rev. 84:101974. doi: 10.1016/j.cpr.2021.101974 33497921

[B78] Trebow E. A. Berkanovic E. Harada P. U. (2015). Outcomes of English- and Spanish-language batterer programs. Partner Abuse 6, 273–297. doi: 10.1891/1946-6560.6.3.273

[B79] Vall B. Sala-Bubaré A. Hester M. Pauncz A. (2021). Impact of intimate partner violence. Int. J. Environ. Res. Public Health 18:5859. doi: 10.3390/ijerph18115859 34072550 PMC8199059

[B80] Whitman M. R. Burchett D. L. Tarescavage A. M. Ben-Porath Y. S. Sellbom M. (2020). Predictive validity of MMPI-2-RF scales. Crim. Justice Behav. 47, 978–995. doi: 10.1177/0093854820918003

